# Pemphigus vulgaris as an immune-related adverse event in recurrent metastatic esophageal squamous cell carcinoma treated with ipilimumab plus nivolumab: a case report and literature review

**DOI:** 10.3389/fimmu.2023.1259071

**Published:** 2023-09-11

**Authors:** Hajime Nakamura, Aika Shionoya, Yohei Arihara, Naotaka Hayasaka, Tomohiro Kubo, Makoto Usami, Shintaro Sugita, Hisashi Uhara, Kohichi Takada

**Affiliations:** ^1^ Department of Medical Oncology, Sapporo Medical University School of Medicine, Sapporo, Japan; ^2^ Department of Dermatology, Sapporo Medical University School of Medicine, Sapporo, Japan; ^3^ Department of Medical Oncology, Steel Memorial Muroran Hospital, Muroran, Japan; ^4^ Department of Surgical Pathology, Sapporo Medical University School of Medicine, Sapporo, Japan

**Keywords:** esophageal squamous cell carcinoma, immune-related adverse events, ipilimumab, nivolumab, pemphigus vulgaris

## Abstract

Ipilimumab plus nivolumab therapy is approved for patients with unresectable advanced esophageal squamous cell carcinoma (ESCC). Although a combination of immune checkpoint inhibitors (ICIs), compared to conventional chemotherapy, can improve overall survival in patients with advanced ESCC, this increases the incidence of immune-related adverse events (irAEs). Here, we describe an ESCC case that developed pemphigus vulgaris (PV), an extremely rare cutaneous irAE, during ipilimumab plus nivolumab treatment. The patient achieved a partial response to treatment. The PV was successfully managed after the cessation of ipilimumab and the use of a topical steroid. We should thus re-treat ESCC with nivolumab monotherapy. In the era of ICIs as standard cancer therapeutics, diagnostic criteria for blistering diseases need to be established to properly manage patients with cutaneous irAEs.

## Introduction

Immunotherapies open new avenues for therapies of many cancers, including gastro-intestinal cancers (1–3). According to results of the CheckMate 648 trial, combination therapy of the immune check point inhibitors (ICIs) of ipilimumab and nivolumab has been introduced for patients with unresectable advanced, recurrent, or metastatic esophageal squamous cell carcinoma (ESCC) as a standard therapy, irrespective of expression levels of tumor cell programmed death-ligand 1 (PD-L1) (4). Combination therapy with ipilimumab plus nivolumab acts as a “double-edged sword” in cancer treatment. That is, the therapy leads to durable anti-tumor effects but also increases incidences of treatment-related adverse events, especially immune-related adverse events (irAEs) (5, 6). Such irAEs are evoked by ICI activities and are characterized by a spectrum distinct from those of cytotoxic chemotherapeutics (6). Oncologists face the diagnosis and management of a variety of irAEs, which range from common to rare and from mild to severe. Cutaneous toxicities, as well as those of the gastrointestinal tract, liver, lung, and endocrine glands, are considered to be common irAEs (7). The CheckMate 648 trial revealed that incidences of rash and pruritus, which were expected irAEs, were more frequent in an ipilimumab plus nivolumab cohort compared to nivolumab plus chemotherapy or chemotherapy-only cohorts (4). Blistering diseases such as bullous pemphigoid (BP), including paraneoplastic pemphigus (PNP) (8–10), and pemphigus vulgaris (PV), are unusual irAEs. In particular, PV is an extremely rare irAE with only three cases reported to date (11–13). Of note, the differential diagnosis of PNP and PV is often complicated. Both PNP and PV are rare autoimmune diseases evoked by the production of autoantibodies due to B-cell hyperactivation (14–16). Noting clinical features and conducting serological examinations are helpful for a differential diagnosis. PNP is known to be mostly induced by hematological malignancies (14). In PV, representative autoantibody targets are desmoglein 1 and 3 (15). In contrast, various targets of autoantibodies exist in PNP, commonly envoplakin and periplakin, including bullous pemphigoid 180 (14). Additionally, pathological findings are useful for the differential diagnosis of PV and PNP. The sites of inflammation induced by autoantibodies of PV and PNP are found in intra-epidermal and basement membrane zones, respectively. Treatment strategies for PV and PNP are local or/and systemic immunosuppression, mainly with corticosteroids (14, 15).

Herein, we present a patient with ESCC who was treated with ipilimumab plus nivolumab and who developed PV as an irAE (PV-irAE). We successfully managed his PV-irAE by the cessation of ipilimumab therapy and with the use of a topical steroid.

## Case presentation

**Figure 1:**
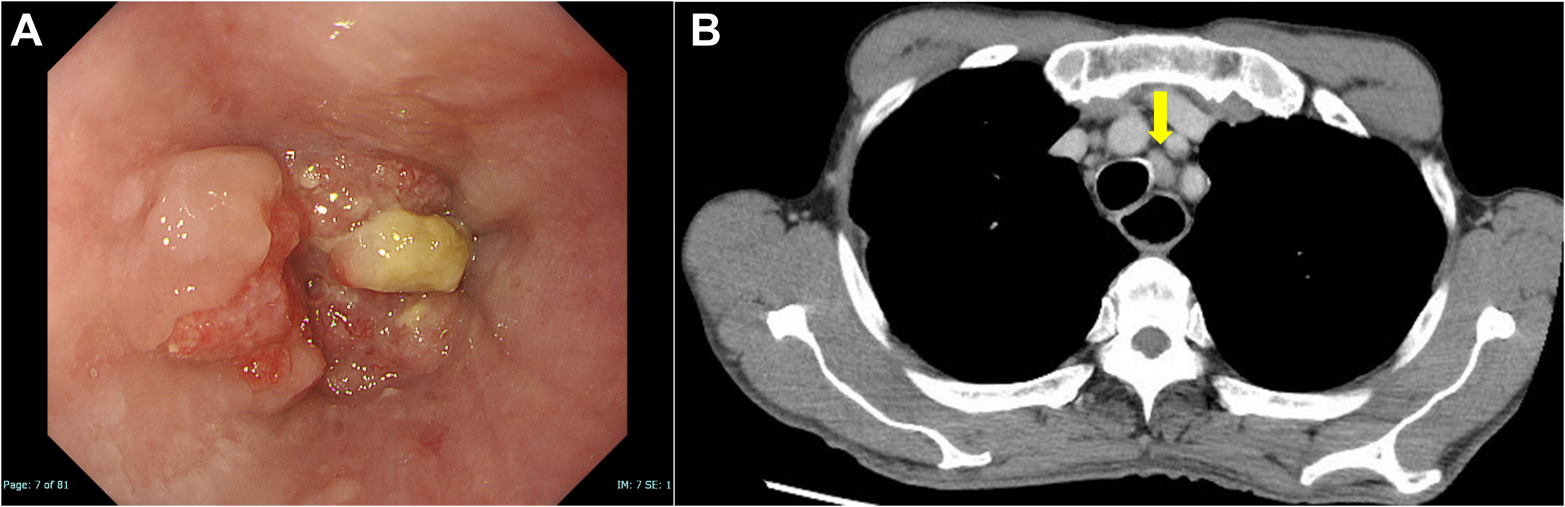
Images of esophageal squamous cell carcinoma on initial diagnosis. **(A)** An upper gastrointestinal endoscopy revealed a squamous cell carcinoma in the middle of the esophagus. **(B)** Enhanced computed tomography revealed mediastinal lymph node metastasis (yellow arrow).

**Figure 2:**
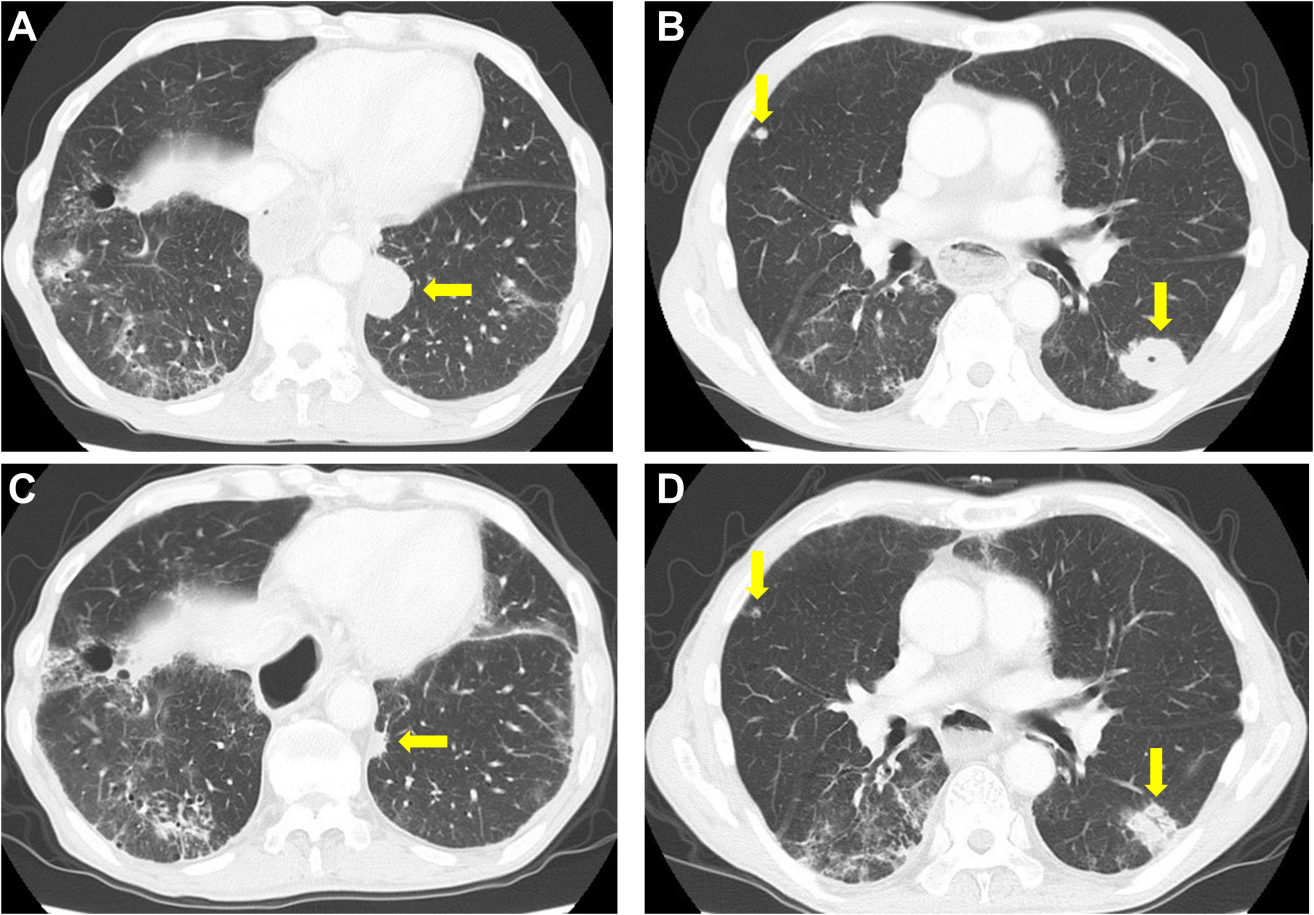
Computed tomography findings. **(A, B)** Pretreatment computed tomography (CT) scan. **(C, D)** CT scan after two cycles of ipilimumab and nivolumab. A decrease in the size of lung tumors was noted. Yellow arrows indicate multiple lung metastases.

**Figure 3:**
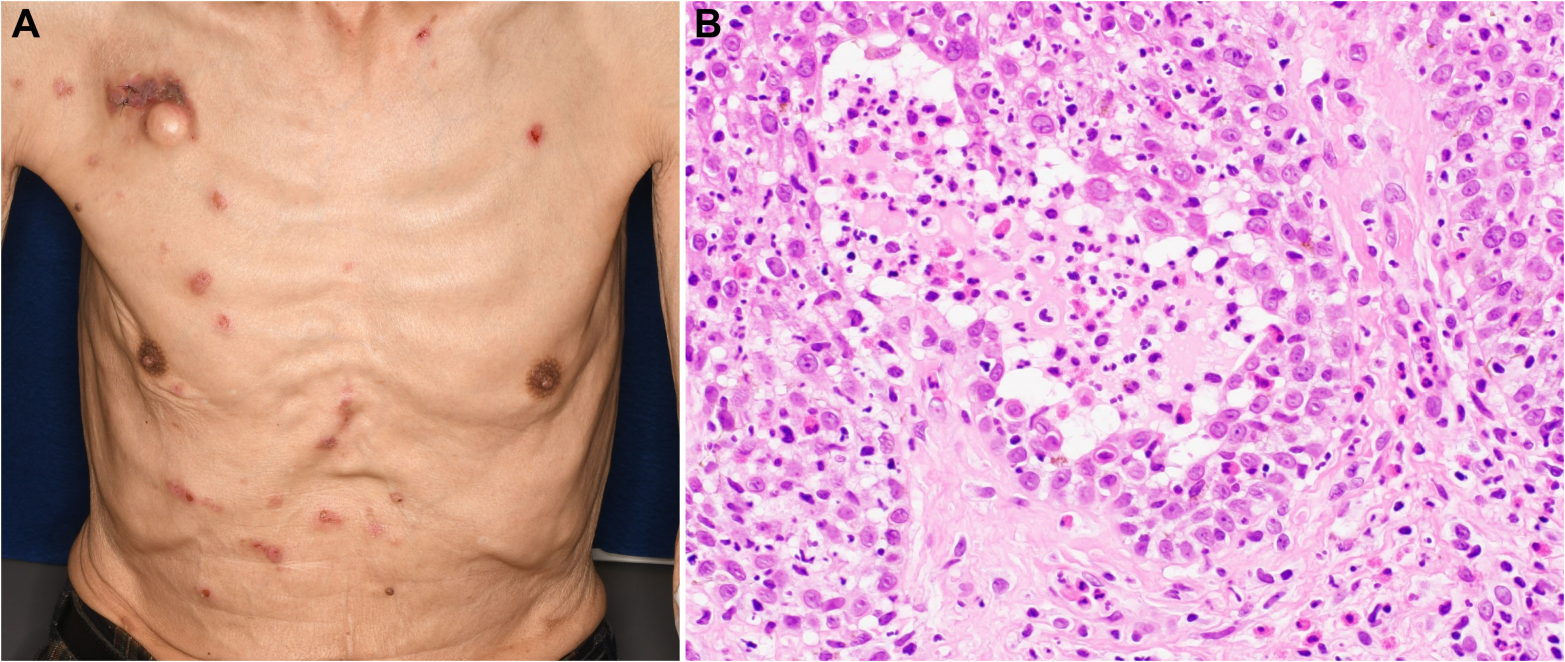
Findings of pemphigus vulgaris. **(A)** Erosions with pruritus on the trunk. **(B)** Hematoxylin–eosin stain (×400) of an intra-epidermal blister demonstrating acantholysis and the loss of keratinocyte intercellular adhesion.

In 2021, a 73-year-old male presented with progressing dysphagia. Upper gastrointestinal endoscopy revealed an advanced circular cancer in the middle of the esophagus (**Figure 1A**). Enhanced computed tomography (CT) revealed mediastinal lymph node metastasis (**Figure 1B**). The patient was diagnosed with ESCC Stage III (T3N1M0) and was treated with three cycles of combination chemotherapy consisting of docetaxel, nedaplatin, and fluorouracil followed by a complete resection (17). He subsequently achieved a pathological complete response. In August 2022, a year after surgery, follow-up CT revealed multiple lung metastases (**Figures 2A, B**). While treatment was planned for his ESCC, the patient became infected with COVID-19. Withholding cancer treatment, the patient was successfully treated with molnupiravir. Subsequently, we initiated ipilimumab plus nivolumab treatment in September 2022. After two cycles of ICI combination therapy (86 days from the first ICI dose), erosions with pruritus developed on the patient’s trunk, especially around the implanted central venous port (**Figure 3A**). Skin swabs were negative for herpes simplex virus and fungus. The cutaneous lesions were initially suspected to be impetigo. Three days after starting topical and oral antibiotics, the patient developed blisters and the number of skin lesions had gradually increased. The patient did not have any mucosal lesions. We performed a skin biopsy and undertook serological examinations to look for immunological cutaneous disorders, including irAEs. A stained histological section of the skin biopsy specimen showed suprabasal epidermal acantholysis and clefting, and blister formation was noted. Blister cavities contained inflammatory cells, including eosinophils and rounded acantholytic cells (**Figure 3B**). Serological tests identified anti-desmoglein 3 antibody but not anti-desmoglein 1 and BP180NC16a antibodies. Direct immunofluorescence did not reveal any deposition of IgG, A, and M, or C3. Based on cutaneous manifestations, pathological findings, and positive anti-desmoglein 3 antibody, we diagnosed Grade 1 PV-irAE. After withholding ICIs, we commenced treatment of the PV-irAE with topical betamethasone. Blisters crusted within a week and most of the skin lesions and pruritus improved by a month after the initiation of betamethasone therapy. Thereafter, we stopped treatment with topical steroid. Despite ceasing cancer therapy, the combined ICI treatment led to a durable and partial response (**Figures 2C, D**). Sixty-two days after the cessation of ICIs, we re-introduced nivolumab only for treatment of the ESCC. Twenty-one days after nivolumab re-treatment, the patient presented with Grade 3 pneumonitis as an irAE. One mg/kg prednisolone was intravenously administered. However, the patient’s performance status worsened and he needed continuous oxygen therapy. After enduring six months of complicated PV-irAE, the patient was transferred to a palliative care hospital after he declined further aggressive treatment for his ESCC.

## Discussion

Immune checkpoint inhibitor combination therapies have now been approved for several cancers and therefore their frequency of use is increasing. As mentioned previously, combination ICI therapies can suppress tumor growth and show durable responses; however, such therapies can evoke various irAEs in systemic organs. Oncologists often encounter many patients with cutaneous irAEs, consisting of a wide variety of diseases of varied severities (7). Accurate diagnoses of cutaneous irAEs are necessary for their treatment. The successful management of irAEs is necessary to achieve good clinical outcomes and improve patients’ quality of life.

We have summarized PV-irAE cases reported in the literature, including our case (**Table 1**). The timing of PV-irAE onset from the first ICI dose varied and symptoms were relatively mild. Notably, PV-irAE could be controlled with topical steroids, and had less effect on the prognosis compared to PNP and BP associated with ICI (9, 16, 18). If PV-irAE is severe, oral prednisone is a treatment option (12).

**Table 1:** Cases with pemphigus vulgaris during immune check point inhibitor treatment.

Case	Age (y) /Sex	Cancer Type	ICI	Response to ICI	Days from the first ICI dose	Symptoms	Treatment	Prognosis*
1^11)^	68/M	Urothelial ca	Nivolumab	NA	188	mild	Oral prednisone	alive
2^12)^	95/M	Cutaneous SCC	Cemiplimab	CR	147	severe	Oral prednisone topical steroid	alive
3^13)^	56/F	Melanoma	Ipilimumab	NA (maintenance)	1	mild	Topical triamcinolone	alive
Our case	73/M	ESCC	Ipilimumab + Nivolumab	PR	86	mild	Topical betamethasone	6 Mo (died)

*: survival time from the diagnosis of cutaneous disease.

ca, carcinoma; CR, complete response; ESCC, esophageal squamous cell carcinoma; ICI, immune checkpoint inhibitor; M, male; Mo, month; NA, not applicable; PD, progressive disease; PNP, paraneoplastic pemphigus; PR, partial response; PV, pemphigus vulgaris; SCC, squamous cell carcinoma.

According to a literature review and our experience, if patients develop blisters during ICI treatment, treating physicians should be mindful of the possible development of cutaneous irAEs. Collaboration between oncologists and dermatologists should be encouraged to manage the development of blistering diseases (16). Dermatologists should also consider performing a skin biopsy to avoid wasting time and inappropriate treatments.

In regard to the case presented here, ipilimumab may have been the cause of the PV since its cessation led to an improvement in symptoms. In addition, nivolumab re-administration did not evoke PV-irAE. Compared to programmed cell death protein 1/PD-L1 inhibitors, ipilimumab more frequently induces cutaneous irAEs (19). Thus, oncologists should pay more attention to the possible development of cutaneous irAEs, including PV, when prescribing ipilimumab therapy. Blistering diseases in patients with cancer during ICI therapy, including nivolumab administration, often lead to deadly complications. Oncologists therefore need to perform prompt diagnostic and therapeutic workups.

## Conclusion

Our case suggests that ipilimumab may trigger PV-irAE, which can be controlled by topical steroid with an early diagnosis. The establishment of novel diagnostic criteria for blistering diseases is urgently required for patients treated with ICIs.

## Data availability statement

The original contributions presented in the study are included in the article/supplementary material. Further inquiries can be directed to the corresponding author.

## Ethics statement

Written informed consent was obtained from the individual for the publication of any potentially identifiable or data included in this article. Written informed consent was obtained from the participant/patient(s) for the publication of this case report.

## Author contributions

HN: Conceptualization, Data curation, Writing – original draft, Writing – review & editing. AS: Data curation, Writing – review & editing, Investigation. YA: Writing – review & editing, Data curation, Investigation. NH: Writing – review & editing, Data curation, Investigation. TK: Writing – review & editing, Data curation. MU: Data curation, Writing – review & editing. SS: Data curation, Writing – review & editing. HU: Data curation, Writing – review & editing, Conceptualization, Supervision, Writing – original draft. KT: Conceptualization, Data curation, Supervision, Writing – original draft, Writing – review & editing.

## References

[1] Shah MA Kennedy EB Alarcon-Rozas AE Alcindor T Bartley AN Malowany AB. Immunotherapy and targeted therapy for advanced gastroesophageal cancer: ASCO guideline. J Clin Oncol (2023) 41(7):1470–91. doi: 10.1200/jco.22.02331 36603169

[2] Abou-Alfa GK Lau G Kudo M Chan SL Kelley RK Furuse J. Tremelimumab plus durvalumab in unresectable hepatocellular carcinoma. NEJM Evidence (2022) 1(8). doi: 10.1056/EVIDoa2100070 38319892

[3] Oh D-Y Ruth He A Qin S Chen L-T Okusaka T Vogel A. Durvalumab plus gemcitabine and cisplatin in advanced biliary tract cancer. NEJM Evidence (2022) 1(8). doi: 10.1056/EVIDoa2200015 38319896

[4] Doki Y Ajani JA Kato K Xu J Wyrwicz L Motoyama S. Nivolumab combination therapy in advanced esophageal squamous-cell carcinoma. N Engl J Med (2022) 386(5):449–62. doi: 10.1056/NEJMoa2111380 35108470

[5] Raschi E Gatti M Gelsomino F Ardizzoni A Poluzzi E De Ponti F. Lessons to be learnt from real-world studies on immune-related adverse events with checkpoint inhibitors: A clinical perspective from pharmacovigilance. Target Oncol (2020) 15(4):449–66. doi: 10.1007/s11523-020-00738-6 PMC7434791 32725437

[6] Postow MA Sidlow R Hellmann MD. Immune-related adverse events associated with immune checkpoint blockade. N Engl J Med (2018) 378(2):158–68. doi: 10.1056/NEJMra1703481 29320654

[7] Schneider BJ Naidoo J Santomasso BD Lacchetti C Adkins S Anadkat M. Management of immune-related adverse events in patients treated with immune checkpoint inhibitor therapy: ASCO guideline update. J Clin Oncol (2021) 39(36):4073–126. doi: 10.1200/jco.21.01440 34724392

[8] Zumelzu C Alexandre M Le Roux C Weber P Guyot A Levy A. Mucous membrane pemphigoid, bullous pemphigoid, and anti-programmed death-1/ programmed death-ligand 1: a case report of an elderly wOman with mucous membrane pemphigoid developing after pembrolizumab therapy for metastatic melanoma and review of the literature. Front Med (Lausanne) (2018) 5:268. doi: 10.3389/fmed.2018.00268 30320114 PMC6170650

[9] Yatim A Bohelay G Grootenboer-Mignot S Prost-Squarcioni C Alexandre M Le Roux-Villet C. Paraneoplastic pemphigus revealed by anti-programmed death-1 pembrolizumab therapy for cutaneous squamous cell carcinoma complicating hidradenitis suppurativa. Front Med (Lausanne) (2019) 6:249. doi: 10.3389/fmed.2019.00249 31750309 PMC6848154

[10] Kawsar A Edwards C Patel P Heywood RM Gupta A Mann J. Checkpoint inhibitor-associated bullous cutaneous immune-related adverse events: a multicentre observational study. Br J Dermatol (2022) 187(6):981–7. doi: 10.1111/bjd.21836 35976170

[11] Ito M Hoashi T Endo Y Kimura G Kondo Y Ishii N. Atypical pemphigus developed in a patient with urothelial carcinoma treated with nivolumab. J Dermatol (2019) 46(3):e90–e2. doi: 10.1111/1346-8138.14601 30168864

[12] Buquicchio R Mastrandrea V Strippoli S Quaresmini D Guida M Filotico R. Case report: autoimmune pemphigus vulgaris in a patient treated with cemiplimab for multiple locally advanced cutaneous squamous cell carcinoma. Front Oncol (2021) 11:691980. doi: 10.3389/fonc.2021.691980 34540666 PMC8444988

[13] Schoenberg E Colombe B Cha J Orloff M Shalabi D Ross NA. Pemphigus associated with ipilimumab therapy. Int J Dermatol (2021) 60(8):e331–3. doi: 10.1111/ijd.15405 33410503

[14] Paolino G Didona D Magliulo G Iannella G Didona B Mercuri SR. Paraneoplastic pemphigus: insight into the autoimmune pathogenesis, clinical features and therapy. Int J Mol Sci (2017) 18(12):2532. doi: 10.3390/ijms18122532 29186863 PMC5751135

[15] Ellebrecht CT Maseda D Payne AS. Pemphigus and pemphigoid: from disease mechanisms to druggable pathways. J Invest Dermatol (2022) 142(3 Pt B):907–14. doi: 10.1016/j.jid.2021.04.040 PMC8860856 34756581

[16] Merli M Accorinti M Romagnuolo M Marzano A Di Zenzo G Moro F. Autoimmune bullous dermatoses in cancer patients treated by immunotherapy: a literature review and Italian multicentric experience. Front Med (Lausanne) (2023) 10:1208418. doi: 10.3389/fmed.2023.1208418 37547602 PMC10400335

[17] Ohnuma H Sato Y Hayasaka N Matsuno T Fujita C Sato M. Neoadjuvant chemotherapy with docetaxel, nedaplatin, and fluorouracil for resectable esophageal cancer: a phase II study. Cancer Sci (2018) 109(11):3554–63. doi: 10.1111/cas.13772 PMC6215867 30137686

[18] Chen WS Tetzlaff MT Diwan H Jahan-Tigh R Diab A Nelson K. Suprabasal acantholytic dermatologic toxicities associated checkpoint inhibitor therapy: a spectrum of immune reactions from paraneoplastic pemphigus-like to Grover-like lesions. J Cutan Pathol (2018) 45(10):764–73. doi: 10.1111/cup.13312 29943453

[19] Muntyanu A Netchiporouk E Gerstein W Gniadecki R Litvinov IV. Cutaneous Immune-Related Adverse Events (irAEs) to Immune Checkpoint Inhibitors: a dermatology perspective on management. Cutan Med Surg (2021) 25(1):59–76. doi: 10.1177/1203475420943260 32746624

